# Children’s perspectives on integrated services: every child matters in policy and practice

**Published:** 2012-06-15

**Authors:** K. Viktoria Stein

**Affiliations:** Institute of Social Medicine, Centre for Public Health, Medical University of Vienna, Rooseveltplatz 3, A-1090 Vienna, Austria, E-mail: katharina.v.stein@meduniwien.ac.at

‘Children’s perspectives on integrated services’ tackles a paramount issue which is still disregarded in many countries because the key proponents do not have a strong voice in our societies: children and youth. Mary Kellett compensates for that by describing the measures taken in England to create integrated services for children and young people, encompassing not only health and social care, but every area children come in contact with during their lives. In order to underline the inclusive approach of the author, every chapter is supplemented by children’s perspectives and research findings reported by children and youth. As such, this book expands the scope of integrated care for children and young people by acknowledging that it takes more than health and education to develop fully, in accordance with one’s talents and preferences.

In the first part of the book, the historical and theoretical background is delineated. Chapter 2 gives a comprehensive overview of the development of child services in the UK from the first Poor Law in 1388 to the ‘Every Child Matters’ (ECM) approach and the Children’s Act 2004. Even though the focus is on the UK and England, the story will read similar for many European countries: beginning with the establishment of the modern health and social care systems in the 20^th^ century, ever more agencies and organizations were tasked with the care for children which led to mismanagement, poor coordination, lack of communication and inadequately trained professionals. In turn, these failings caused the tragic death of individual children, which became the motive and cause for reforms and reports to improve the services. In the UK, this ultimately culminated in the Children’s Act 2004 and in the ‘Every Child Matters’ (ECM) approach. The success of and experiences with this ECM approach is hence the main focus of the book.

The historical evolution of child services is complemented by a brief description of different schools of thought, namely from economics, sociology and psychology on the roles and responsibilities of children while growing up. This discourse is dominated by different views on power and by an evolution of discourses from the needs, via the rights to the quality of life of children. The author sets these theoretical concepts into the wider context of the political landscape from the post-WWII era via Thatcher and New Labour to the present day ambiguity. While the historical background gives a comprehensive insight into the development of child services, the political and theoretical underpinnings stay behind their potential to explore the influence of scientific concepts on political and societal decisions.

Part II encompasses the wide array of services and aspects to be considered when dealing with children, detailing the latest changes and reforms that have been introduced for the various professions and service providers: from education, health and social services to safeguarding, supporting families and considering children as active participants of society. Focusing on the child’s perspectives and wishes and actively involving them in the decision-making about their care needs is the underlying rationale that runs throughout the reform efforts and the newly established integrated services under the ECM concept and the Children’s Act 2004. The ECM concept established five principles which are to be followed and incorporated into the services organized around the child: being healthy, staying safe, enjoying and achieving, making a positive contribution, and achieving economic well-being.

Chapters 4–6 cover social work, education and the health services, respectively, always including excerpts of children’s views. With the establishment of the integrated child centers and the lead professional, the necessity for multi-agency cooperation and interdisciplinary teamwork arose. This in turn necessitated a better and more coordinated professional training and education for the service providers involved.

A major challenge was the abolition of a distinction between the different areas of care offered to children (education, childcare, social services and health) and the creation of a centralized organization starting with the Department for Children, Schools and Families on the national level in 2007 down to the Children’s Trusts on local level. The idea was to reduce the number of people involved in servicing children and make it easier for them and their families to know their contact persons. ECM also propagated the active involvement of children in the decision-making process about their care, which is exemplified by simply asking them about their experiences and wishes or the Pupil Voice initiative. By building the service centers around the child, however, the risk arose that the time organized for children and the already tight schedules extended even into early childhood and, via the extended schools model, into the leisure time as well.

ECM is intended for every child in the UK, however, special focus is laid on those most vulnerable: children with disabilities or chronic diseases, children being poor, being abused or otherwise marginalized, children with migrational backgrounds or asylum-seekers, and looked-after children (Chapters 4–10). Most of the developed programmes targeting these children hence also take a look at the family situation, offering support and assistance to the parents as well. The rationale is that prevention is better than cure and that a safe and stable familial environment will produce healthy, safe and happy children. Key components often are educational measures, organizing leisure time activities and creating a platform for exchange for children and parents with similar needs. Many of these activities are provided by third sector organizations (Chapter 9), which were actively included by New Labour into the ECM concept. The idea was that the third sector is more flexible and trusted and its value-driven ethos may provide easier and access to local communities than state agencies. Commissioning services from the third sector hence became a vital part of ECM. However, a third sector organization does not per se deliver better services than a private or public sector one and the shift to actively commissioning third sector services via short-term contracts made them more dependent and vulnerable.

Finally, part III of the book ties the knot, describing methods of active involvement of children and children as researchers. Throughout the book, emphasis is laid on presenting children’s views and in Chapter 12, it is described, how these views were collected. Together with children the author developed research methods, based on the scientific principles of reproducibility and evidence-generation, which were suitable for children to conduct: including interviews, surveys and data analyses, as well as more creative ones, using photography, building stones or drawings. Additionally, ECM itself has created various tools to activate children and make their opinions heard. Kellett makes a strong point for these forms of active participation of children to give their voices more strength and credibility. After all, we still live in societies where children’s voices usually are regarded as a nuisance or as not being taken seriously at best. Albeit, as the author doesn’t tire to point out, the UN Children’s Rights Charter (UNCRC) requires governments and societies to grant children the same rights as adults, along with the necessary protection (Chapter 11).

The book gives a comprehensive and impressive overview of the reorganization and reform process initiated by ECM and makes a strong point to integrate all services concerned with children. At the same time, it doesn’t fall short of describing the pitfalls and dangers of the approach. Integration of services here means much more than merely inter-sector cooperation or the creation of integrated structures. It propagates an understanding that one does not work without the other: it takes a healthy, safe and supportive environment to enable children to learn and grow up to be responsible and self-respecting adults. There is no health without education, no development without encouragement and respect. Even though the book describes the English situation, lessons can be learned for other countries and all professions, and one can only hope that as many professionals and decision-makers as possible read this book.

